# Ectopic Fat and Insulin Resistance: Pathophysiology and Effect of Diet and Lifestyle Interventions

**DOI:** 10.1155/2012/983814

**Published:** 2012-05-24

**Authors:** M. Snel, J. T. Jonker, J. Schoones, H. Lamb, A. de Roos, H. Pijl, J. W. A. Smit, A. E. Meinders, I. M. Jazet

**Affiliations:** ^1^Departments of Endocrinology & Metabolism and General Internal Medicine, Leiden University Medical Center, P.O. Box 9600, 2300 RC Leiden, The Netherlands; ^2^Walaeus Library, Leiden University Medical Center, P.O. Box 9600, 2300 RC Leiden, The Netherlands; ^3^Department of Radiology, Leiden University Medical Center, P.O. Box 9600, 2300 RC Leiden, The Netherlands

## Abstract

The storage of triglyceride (TG) droplets in nonadipose tissues is called ectopic fat storage. Ectopic fat is associated with insulin resistance and type 2 diabetes mellitus (T2DM). Not the triglycerides per se but the accumulation of intermediates of lipid metabolism in organs, such as the liver, skeletal muscle, and heart seem to disrupt metabolic processes and impair organ function. We describe the mechanisms of ectopic fat depositions in the liver, skeletal muscle, and in and around the heart and the consequences for each organs function. In addition, we systematically reviewed the literature for the effects of diet-induced weight loss and exercise on ectopic fat depositions.

## 1. Introduction

The amount of people with obesity has increased dramatically over the past decades to an estimated number of 400 million adults worldwide with a projected 700 million in 2015 [1]. Obesity predisposes to the development of insulin resistance, type 2 diabetes mellitus (T2DM), and cardiovascular disease (CVD) [2–7]. However, about 30% of obese men and women are metabolically healthy [8], that is, do not have hypertension, dyslipidemia, or disturbances in glucose metabolism. Vice versa, these metabolic abnormalities occur in 20–30% of normal weight people.

Adipose tissue consists of adipocytes and the so-called stromal-vascular fraction that encompasses blood vessels and stroma with macrophages. Adipose tissue has the unique capacity to store large amounts of energy in the form of triglycerides (TG). For a long time, it has been presumed that this was the only function of adipose tissue. However, adipose tissue (i.e., adipocytes and the stromal vascular fraction) acts as an endocrine organ by secreting various hormones and cytokines (also referred to as adipokines) with effects on glucose and lipid metabolism and energy homeostasis [9]. It now appears that in those obese subjects that develop insulin resistance, adipose tissue dysfunction plays a role. Adipose tissue dysfunction is characterized among others by large adipocytes and secretion of adipokines with a proinflammatory profile ultimately leading to (among others) ectopic fat deposition [10]. Ectopic fat is defined as storage of TG in tissues other than adipose tissue, that normally contain only small amounts of fat, such as the liver, skeletal muscle, heart, and pancreas. Ectopic fat can interfere with cellular functions and hence organ function and is associated with insulin resistance.

The aim of this paper is to discuss the pathophysiology of adipose tissue dysfunction and ectopic fat deposition. We will elaborate on the effect of ectopic fat deposition on the cellular level as well as on the organ level for those organs involved in the insulin-resistant pathogenesis of T2DM (liver and skeletal muscle cell) and on the heart (because of the strongly increased risk for coronary heart disease in T2DM). Since there is uncertainty to date as to whether pancreatic steatosis is causally related to beta-cell dysfunction [11–13], and because it is not subject of investigation in our own studies, we will not discuss fat accumulation in the pancreas. Finally, because diet and exercise are the mainstays of therapy for obesity and T2DM, we systematically reviewed the literature for the effect of diet and or exercise on diminishing ectopic fat stores and restoring insulin sensitivity in obesity and T2DM.

## 2. Methods Systematic Review

The following databases were searched: PubMed (1949 to January 2012), EMBASE (OVID-version, 1980 to January 2012), Web of Science (1945 to January 2012), and Cochrane Library (1990 to January 2012). The search strategy consisted of the AND combination of three main concepts:

1. type 2 diabetes mellitus, Obesity, or insulin resistance;
2. weight loss, diet, or exercise;
3. ectopic fat.

For these three concepts, all relevant keyword variations were used. References were limited to human studies, adults, written in English or Dutch. In addition, only studies that used techniques that can quantify the amount of lipid accumulation and measured insulin sensitivity were included. Studies using surrogate markers for lipid accumulation (e.g., alanine aminotransferase (ALT) or aspartate aminotransferase (AST) as a proxy for hepatic steatosis) were excluded. Hypocaloric diets are defined as containing less calories than required for energy demands and usually contain 1000–1200 kcal/day. Very low-calorie diets (VLCDs) typically contain less than 800 kcal/day. See the appendix for literature search details.

## 3. Adipose Tissue Dysfunction

The cause for adipose tissue dysfunction and ectopic fat storage is largely unknown. Blüher [10] recently proposed a model in which genetic, environmental, and behavioral factors are involved in excess energy intake and decreased physical activity on one hand as well as physiologic versus pathologic fat accumulation on the other hand. The physiologic response to excess energy intake and less expenditure would be an increase in fat cell number as well as fat cell size (hyperplasia of adipose tissue) in the subcutaneous compartment. These fat cells would function normally and be insulin sensitive. On the contrary, a pathophysiologic response would be adipocyte hypertrophy.

Human adipocytes can grow up to ~20 fold in diameter and several thousand-fold in volume [14]. However, when adipocytes become too large, stress signals will be released. For example, hypoxia can occur when vascularisation is inadequate for the expanded adipose tissue. In addition, endoplasmatic reticulum (ER) stress [15], either induced by hypoxia or nutrient excess, leads to an unfolded protein response (UPR) [16]. In the ER, proteins are translated, folded, and assessed for quality before release. With ER stress the number of misfolded proteins increases and triggers the UPR. The UPR induces genes involved in assembling, folding, modifying, and degrading proteins to alleviate ER stress and triggers the activation of stress and inflammatory pathways and the production of cytokines and chemokines that interfere with the insulin signaling pathway.

Dysfunctional adipocytes produce more inflammatory adipokines and cytokines than functional adipocytes. In addition, stressed adipocytes attract immune cells (among which macrophages) into the stromal vascular fraction. The physiological role of infiltrating macrophages is probably debris clearing (ER stress can eventually lead to premature adipocyte apoptosis). Eventually however, a positive feedback cycle is formed in which infiltrating macrophages recruit more immune cells, and a state of chronic inflammation is induced. Some of the cytokines and adipokines produced interfere with adipocyte differentiation, others with insulin signaling. Some, like TNF-*α* and IL-6, impair adipocyte differentiation, reduce lipid accumulation, and increase adipocyte lipolysis.

The latter is on top of the poor ability of stressed and hypertrophic adipocytes to take up and release free fatty acids (FFAs). This induces a redirection of lipids towards peripheral tissues, including skeletal muscle, liver, pancreas, and heart: ectopic fat deposition.

**Figure 1:**
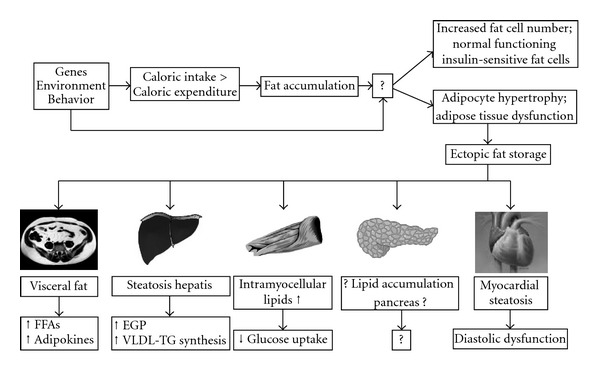
Ectopic fat depositions. Gene-environment interactions might be involved both in increased energy intake and decreased physical activity as well as in the response of adipose tissue to the ensuing increased energy balanced. Predisposed subjects will elicit a pathophysiologic response leading to adipocyte hypertrophy. This will lead to an inflammatory response that ultimately leads to ectopic fat deposition. The consequences of ectopic fat are organ specific as depicted in this figure. The effect of ectopic fat on the cellular level is different and depicted in Figure 2. FFAs: free fatty acids; EGP: endogenous glucose production; VLDL-TG: very low-density lipoprotein-triglyceride.

 If in these tissues, lipid supply exceeds oxidative capacity intracellular lipid accumulation occurs, and organ function might be impaired (Figure 1).

## 4. Cellular Mechanisms of Ectopic Fat-Induced Organ Dysfunction

**Figure 2:**
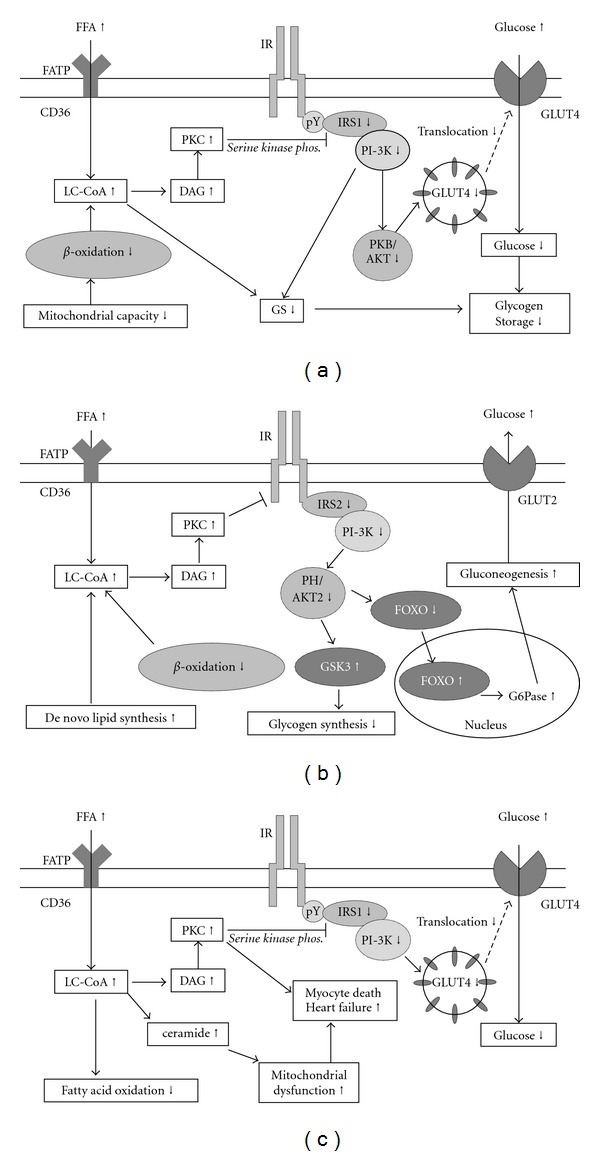
(a) cellular processes leading to insulin resistance in the skeletal muscle cell; (b) cellular processes leading to insulin resistance in the liver cell; (c) cellular processes in the cardiomyocyte leading to myocardial dysfunction.

The consequences of ectopic fat accumulation depend on the specific organ. However, the mechanisms leading to the disruption of organ function are quite similar at the cellular level (Figures 2(a)–2(c)).

Firstly, it has to be noted that lipids can be dispersed intercellularly or accumulate intracellularly. Intercellular lipid accumulation might impair organ function via paracrine effects of the released adipokines. However, it is intracellular lipid accumulation that is associated with decreased insulin sensitivity.

Free fatty acids are taken up by the cell via specific transport proteins in the cell membrane (CD36, fatty acid transport protein (FATP), along with passive diffusional uptake [17]. Inside the cell, fatty acid binding protein (FABPc) is the most important cytosolic protein for guiding long-chain fatty acids in the cell to places of oxidation or esterification. Long-chain fatty acyl-CoA is taken up by the mitochondria via carnitine-palmitoyl transferase 1 (CPT1). Inside the mitochondria, *β*-oxidation and further degradation in the tricarboxylic acid cycle takes place. Therefore, intracellular lipid accumulation occurs as a consequence of continuous oversupply of FFAs (caused by enhanced lipolysis, adipocyte dysfunction) together with an impairment in FFA oxidation in the mitochondria.

It appears that not the FFAs themselves but rather metabolites like long-chain acyl-CoA (LC-CoA), diacylglycerol (DAG), and ceramides are deleterious for the cell. These fatty acid metabolites induce a sustained activation of serine/threonine kinases such as protein kinase C (PKC) isoforms, IKB-kinase-*β* and Jun N-terminal kinase, which phosphorylate insulin-receptor substrates (IRS) on serine residues [18]. The subsequent defects in insulin signaling lead to a decrease in cellular function that depends on the cell type.  Figure 2 describes the effects of intracellular lipid accumulation in skeletal muscle, liver, and cardiomyocytes [18, 19]. The explanation for the various organs is described in the following paragraphs and is the explanation of Figures 2(a) to 2(c).

FFAs are taken up by the skeletal muscle cell mainly by protein-mediated membrane transport (CD36, fatty acid transport protein (FATP)), along with passive diffusional uptake. Long-chain fatty acyl-CoA is taken up by the mitochondria via carnitine-palmitoyl transferase 1 (CPT1) [17]. Inside the mitochondria *β*-oxidation and further degradation in the tricarboxylic acid cycle takes place. When there is an oversupply of FFAs and/or an impairment in mitochondrial *β*-oxidation exist, LC-CoA accumulates and is broken down to intermediates like DAG and ceramide. These fatty acid metabolites induce a sustained activation of serine/threonine kinases such as protein kinase C (PKC) isoforms, IKB-kinase-*β* and Jun N-terminal kinase, which phosphorylate insulin-receptor substrate (IRS) 1 on serine residues [18]. Serine-phosphorylated forms of IRS1 cannot associate with and activate phosphatidyl-inositol-3-kinase (PI3K), resulting in a decreased glucose transporter 4 (GLUT4) regulated glucose transport over the cell membrane. FATP: fatty acid transport protein; FFA: free fatty acid; LC-CoA: long-chain fatty acid-CoA; DAG: diacylglycerol; PKC: protein kinase C; IR: insulin receptor; IRS: insulin receptor substrate; PI3K: phosphatidylinositol 3-kinase; PKB/AKT: protein kinase B/AKT; GLUT4: glucose transporter protein 4; GS: glycogen synthase.

The mechanism behind hepatic TG accumulation and the development of hepatic insulin resistance are similar to that described for skeletal muscle [18, 20]. An increase in DAG in hepatic cells leads to activation of PKC, leading to decreased insulin receptor kinase activity and subsequently lower insulin-stimulated IRS2 tyrosine phosphorylation and lower IRS2-associated PI3K-activity which results in reduced insulin stimulation of glycogen synthase activity. This ultimately leads to decreased insulin-stimulated hepatic glucose uptake and reduced insulin suppressibility of hepatic glucose production. Furthermore, reduced activity of AKT2, a protein kinase downstream of IRS and PI3K, results in decreased phosphorylation of the forkhead box O (FOXO) transcription factor, allowing it to enter the nucleus and activate the transcription of the rate-controlling enzymes of gluconeogenesis. The result is increased hepatic glucose production and decreased hepatic glucose uptake, which both contribute to increased plasma glucose levels. FATP: fatty acid transport protein; FFA: free fatty acid; LC-CoA: long-chain fatty acid-CoA; DAG: diacylglycerol; PKC: protein kinase C; IR: insulin receptor; IRS: insulin receptor substrate; PI3K: phosphatidylinositol 3-kinase; GSK3: glycogen synthase kinase 3; FOXO: forkhead box protein O; GLUT2: glucose transporter protein 2.

In the heart, increased FFA supply and decreased oxidation lead to lipid intermediates that impair insulin signaling, and hence GLUT-4 involved glucose uptake. In addition, lipid-induced PKC activation might be involved in premature adipocyte apoptosis and heart failure. Finally, accumulation of lipids increase DAG and ceramide levels inducing a vicious cycle in which a further impairment in mitochondrial fatty acid oxidation occurs that also has negative effects on myocardial function. FATP: fatty acid transport protein; FFA: free fatty acid; LC-CoA: long-chain fatty acid-CoA; DAG: diacylglycerol; PKC: protein kinase C; IR: insulin receptor; IRS: insulin receptor substrate; PI3K: phosphatidylinositol 3-kinase; GLUT4: glucose transporter protein 4.

## 5. Skeletal Muscle

### 5.1. Intramyocellular Lipids and Peripheral Insulin Resistance

Accumulation of intramyocellular lipids (IMCLs) as measured by skeletal muscle biopsies or ^1^H-magnetic resonance spectroscopy is associated with insulin resistance [21–26] and T2DM. However, it is not synonymous with the condition given the fact that endurance-trained athletes, who are highly insulin sensitive, also have a high IMCLs content [26]. Rather, the capacity to oxidize IMCLs determines whether they represent a physiological or a pathological role [27]. In endurance-trained athletes IMCLs are an adaptive response, the IMCLs serve as a readily available energy source. The close proximity of lipid droplets to the mitochondria supports this hypothesis. In these athletes, IMCLs are not deleterious because of the increased capacity to oxidize lipids. In insulin-resistant and/or T2DM patients, the increased IMCLs stores are the result of increased FFA availability and impaired fatty acid oxidation [28–30]. The latter leads to accumulation of lipid intermediates with the ascribed toxic effects on insulin signaling (Figure 2). Hence, the ratio between IMCLs and fat oxidative capacity is a better marker for insulin resistance than IMCLs alone.

The fact that fatty acid oxidation is impaired in insulin-resistant states led to the speculation that mitochondrial dysfunction is the cause of IMCLs accumulation and the ensuing insulin resistance. Indeed, a decreased mitochondrial density and/or function has been reported in insulin-resistant offspring of T2DM patients [31–33] and T2DM patients [34–37]. However, three of these studies reported decreased mitochondrial function at normal IMCLs levels [35–37] suggesting that impaired mitochondrial function is not a prerequisite for IMCLs accumulation. Rather, it might be that mitochondrial dysfunction is the consequence of the increased amount of fatty acid metabolites, for example, via the formation of lipid peroxides [38]. In that case it might be that the lipid-induced mitochondrial dysfunction induces progressive deterioration of oxidative capacity and further accumulation of lipid intermediates in the skeletal muscle cell. Further investigations are warranted to elucidate which one is cause or consequence: IMCLs or mitochondrial dysfunction. In this paper, we focus on IMCLs and will not elaborate on mitochondrial function.

### 5.2. Effect of Diets on IMCLs Accumulation (Table 1)

When assessing the effects of diet on insulin sensitivity and IMCLs content both severity of the caloric restriction, duration of the intervention, amount of weight loss and perhaps severity of insulin resistance and duration of T2DM have to be taken into account. From the available literature (Table 1), it is difficult to draw definite conclusions as to which of these parameters has the greatest effect on improving insulin sensitivity and IMCLs [39–45].

For example, moderate weight reduction (3–11 kg) with hypocaloric diets showed no effect on insulin sensitivity or on IMCLs content in normal glucose tolerant (NGT) obese subjects [39, 42, 44–46] or T2DM patients [41, 43] in 3 studies. However, a recent study did show an increase in insulin-stimulated glucose disposal along with a decrease in IMCLs content following around 8 kg weight reduction with a hypocaloric diet (−500 to –40 kcal/day based on food diaries) in elderly obese subjects with impaired fasting glucose or impaired glucose tolerance [47]. Maybe the low fat content of the diet accounts for the observed difference as compared to the other 3 studies. Alternatively, the pathogenesis of impaired glucose metabolism (and hence its reversal) might be different in older as compared to younger subjects.

The amount of caloric restriction seems to be important since moderate weight reduction with a low-calorie diet [45] or a very low-calorie diet [48] significantly improved insulin sensitivity although it had no effect on IMCLs in obese NGT subject. One study using a VLCD showed not only an improvement in insulin sensitivity but also a decrease in IMCLs, even after 6 days of the VLCD, in obese and T2DM patients [49]. It should be noted; however, that IMCLs were measured in soleus muscle with ^1^H-MRS, whereas other studies mostly measure IMCLs in the vastus lateralis muscle. In addition, with respect to insulin sensitivity, very high doses of insulin (200 mU/m^2^/min) were used.

Pronounced weight loss (~22 kg) using a VLCD (450 kcal/day, on average 17 weeks duration) in obese insulin-treated T2DM patients not only improves peripheral insulin sensitivity but also decreases IMCLs in skeletal muscle biopsies [50] (Table 1).

### 5.3. Effect of Diet and Exercise on IMCLs Accumulation

Several studies investigated the effect of the addition of exercise to a hypocaloric or VLCD in obese NGT or obese T2DM patients (Table 1). All studies in obese NGT [45, 51, 53], obese impaired glucose tolerant (IGT) [54], or obese T2DM patients [43, 52] consistently showed increased insulin sensitivity with no differences between diet-only or diet with exercise group (with the exception of [45] all measured with the hyperinsulinaemic euglycaemic clamps). The effect of adding exercise on IMCLs in these studies was less consistent showing either no effect [45, 51, 53], a decrease [43, 54], or even an increase [52] in IMCLs accumulation.

### 5.4. Effect of Exercise on IMCLs Accumulation

Few studies have addressed the effect of exercise only on IMCLs and insulin sensitivity. Three studies on obese NGT [55] and obese IGT subjects [47, 54] showed an increase in insulin-stimulated glucose disposal despite minimal weight loss (1–3 kg). Two of those studies [47, 55] showed an increase in IMCLs with a decrease in intramuscular ceramide and DAG levels along with increased lipid oxidation rates. The other study showed a decline in IMCLs both in the diet + exercise as well as in the exercise-only group [54].

In 18 T2DM patients (body mass index (BMI) 30 kg/m^2^, HbA1c 7.2%, age 59 years) a 12-week combined aerobic and resistance program had no effect on body weight [56] but improved insulin-stimulated glucose disposal and increased IMCLs along with an increase in insulin-stimulated glucose oxidation and suppression of fat oxidation (Table 1). Interestingly, the same intervention had no effect on peripheral insulin sensitivity and IMCLs in healthy-matched controls.

### 5.5. Conclusion

Moderate weight loss has no effect on IMCLs but can improve peripheral insulin sensitivity if a VLCD is used. Larger weight losses are needed to improve both peripheral insulin sensitivity and deplete IMCLs stores. With respect to the effect of exercise on IMCLs, the story is more complex. Athletes have increased IMCLs stores that serve as a readily available energy supply and training further increases IMCLs in healthy subjects. On the other hand, sedentary insulin-resistant subjects also have increased IMCLs, caused by diminished oxidative capacity. These are associated with increased intramyocellular lipid intermediates that interfere with insulin signaling and other cellular processes. Exercise training in these subjects either increases, decreases, or has no effect on IMCLs accumulation. This is probably associated with the stage of disease (can fatty acid oxidation and/or mitochondrial function be restored) as well as with the intensity of the exercise program and sort (aerobic/resistance) exercise. Overall, studies show that exercise improves peripheral insulin sensitivity. Factors involved are a reduction in lipid intermediates and hence improved insulin signaling and mitochondrial function, increased oxidative capacity and increased capillary blood flow. In addition, contraction and hypoxia activate adenosine monophosphate-activated protein kinase (AMPK) which leads to increased GLUT4 translocation independent of insulin.

## 6. Liver

### 6.1. Intrahepatic Lipids and Insulin Resistance of the Liver

Accumulation of fat in the liver in the absence of excessive alcohol ingestion is referred to as nonalcoholic fatty liver disease (NAFLD). The spectrum of liver abnormalities within this entity ranges from hepatic steatosis with or without mild increases in serum AST/ALT to nonalcoholic steatohepatitis (NASH) with or without fibrosis, cirrhosis, and incidental hepatocellular carcinoma. The worldwide estimated prevalence of NAFLD in the general population is about 20%, with large differences across countries [57]. There is a strong positive correlation between NAFLD and obesity. A cross-sectional study on 1515 severely obese NGT subjects showed some signs of fatty liver changes in almost 90% of people [58]. The majority had hepatic steatosis, but one-third had portal inflammation and fibrosis. A prospective study demonstrated a 4-time increased risk to develop hepatic steatosis in obese persons as compared to controls [59].

Noninvasive methods for measuring hepatic TG content such as ultrasound, CT, and ^1^H-MRS cannot distinguish NAFLD from NASH and fibrosis. A definite diagnosis can only be made by liver biopsy with histologic examination. The cutoff value for abnormal lipid accumulation in the liver has been defined as more than 5% of liver volume or when more than 5% of hepatocytes contain visible intracellular lipids [60]. Two recent studies in respectively a NGT mixed (Hispanic, non-Hispanic, and African American) population [61] and a lean Caucasian population [62] found that the 95th percentile for hepatic TG content was 5.6% and 3%, respectively, using ^1^H-MRS. The Pathology Committee of the NASH Clinical Research Network designed and validated a scoring system of 14 histological features examining liver biopsy findings detailing steatosis, fibrosis, inflammation, and liver cell injury [63]. An NAFLD activity score >5 was universally associated with NASH.

Fat accumulation in the liver is associated with hepatic insulin resistance as well as with peripheral insulin resistance in skeletal muscle and adipose tissue [64–67]. In a large European cohort of 1307 middle-aged NGT subjects, patients with a high fatty liver index (an estimate for hepatic steatosis based on an algorithm including BMI, waist circumference, TG, and gamma-glutamyltransferase) had a lower glucose disposal rate as measured by a euglycaemic hyperinsulinaemic clamp as well as higher FFA levels at the end of the insulin infusion [68]. The latter is suggestive for decreased insulin sensitivity of adipose tissue. Korenblat et al. [64] found a negative correlation between hepatic insulin sensitivity measured by the hyperinsulinaemic euglycaemic clamp and hepatic TG content (measured by ^1^H-MRS) in 42 nondiabetic obese subjects. Indeed, a multivariate linear regression analysis found that hepatic TG content was the best predictor of insulin sensitivity in liver, skeletal muscle, and adipose tissue, independent of BMI and percent body fat. Some claim that the amount of hepatic TG content is directly correlated with the severity of insulin resistance, but this cannot be confirmed by others.

### 6.2. Effect of Diet on Intrahepatic Lipids

Noninvasive techniques like CT and ^1^H-MRS have shown that weight loss by nutritional interventions can result in a large decrease in hepatic TG content in obese and T2DM subjects [41–43, 69–73]. Because of the different populations and differences in baseline hepatic TG content, studies are not well comparable with respect to the individual effect of level of caloric restriction and/or amount of weight reduction on loss of hepatic TG content. Nonetheless, it has been shown that even a relatively small drop in BMI of 3–6% is associated with a considerable reduction in hepatic TG content of 34–40% [42, 70, 71]. The main reduction in hepatic TG content already occurs in the first two weeks of dietary restriction [43, 69]. The percentual decline in hepatic TG content positively correlates with the hepatic TG content at baseline, as patients with a high hepatic TG content at start of the diet lose relatively more TG than patients with low hepatic TG content, with the same amount of weight loss [70, 72]. Only two studies measured both hepatic TG content and hepatic insulin sensitivity with a hyperinsulinaemic euglycaemic clamp in obese NGT [73] and obese T2DM patients, respectively [41]. Both studies found an improvement in hepatic insulin sensitivity that was associated with the decrease in intrahepatic lipids (IHL).

Although the above-mentioned studies clearly show that diet-induced weight loss leads to a decrease in hepatic TG content, the effect of this decrease in hepatic TG content on liver histology (i.e., with liver biopsies) has only been scarcely studied. In obese patients with NASH moderate weight loss over a 12-month period, obtained with dietary advices only, led to an improved steatosis score in 9 of the 15 subjects. The improvement was associated with greater weight loss as compared to the patients that showed no change in steatosis [74]. Another study on 15 patients with obesity and NASH combined a hypocaloric diet with exercise during 12 weeks, while 10 obese subjects with NASH served as controls. The BMI decreased by 3 kg/m^2^ in the intervention group, and the steatosis score decreased from 2.3 to 1.3 (30–50% steatosis to less than 30%), while no changes were observed in the control group [75]. In contrast, severe caloric restriction during 8 months in 41 morbidly obese (BMI 43.3 kg/m^2^) subjects leading to an impressive median weight loss of 34 kg (−27% of BMI) showed normalization of liver architecture in 19 patients [76]. However, they also found an increase in hepatic inflammation and fibrosis in some patients, this was associated with a greater weight loss and elevated FFAs.

### 6.3. Effect of Diet and Exercise on Intrahepatic Lipids

Most studies combine an exercise program with a hypocaloric diet. Since short-term diet alone already reduces IHL, it is difficult to extrapolate whether adding exercise has additional effects [77, 78]. Both in obese as well as in T2DM patients combined diet with exercise interventions led to a reduction of hepatic steatosis. Larson-Meyer studied a diet-only (25% calorie restriction (CR)) versus a diet (12.5% CR) combined with exercise (12.5% CR) obese NGT group. In this 6-month study no additional effect of exercise upon the diet was found at an equal total amount of caloric restriction [45]. Another 6-month study in older sedentary obese subjects also showed no additional effect of exercise on IHL content and insulin sensitivity measured by oral glucose tolerance test (IHL diet: −46 ± 11%, diet + exercise: −45 ± 8%; insulin sensitivity diet: 66 ± 25%, diet + exercise: 68 ± 28%) [79].

### 6.4. Effect of Exercise on Intrahepatic Lipids

Only one study has investigated the influence of exercise alone on hepatic TG content in adult overweight (BMI 27.7 ± 0.5 kg/m^2^) sedentary men [80] and simultaneously measured hepatic insulin sensitivity with a state of the art technique. After a 6-week aerobic exercise program (60–85% of VO_2max⁡_ for a minimum of 20 min. at least three times per week) without significant effect on body weight no changes were found in hepatic TG content as measured by ^1^H-MRS, although both peripheral and hepatic insulin sensitivity (measured by the hyperinsulinaemic euglycaemic clamp) improved.

This is in contrast with a study in obese NGT subjects in which a one-month aerobic exercise training (3 times/week, 3–45/min at max 70% VO_2max⁡_) decreased hepatic TG concentration by 21% at equal body weight. Hepatic insulin sensitivity was not measured, HOMA-IR remained unchanged [81].

Perhaps the amount of IHL at start of the study and/or the severity of the impairment in glucose metabolism accounts for the observed differences.

Interestingly, in 196 overweight, sedentary dyslipidemic subjects an 8-month exercise intervention showed that aerobic exercise is more effective in reducing liver fat and insulin sensitivity (measured by HOMA) than resistance training. A combined resistance and aerobic exercise program was as effective as aerobic exercise on the above-mentioned outcome parameters [82].

## 7. Epicardial and Pericardial Fat

**Figure 3:**
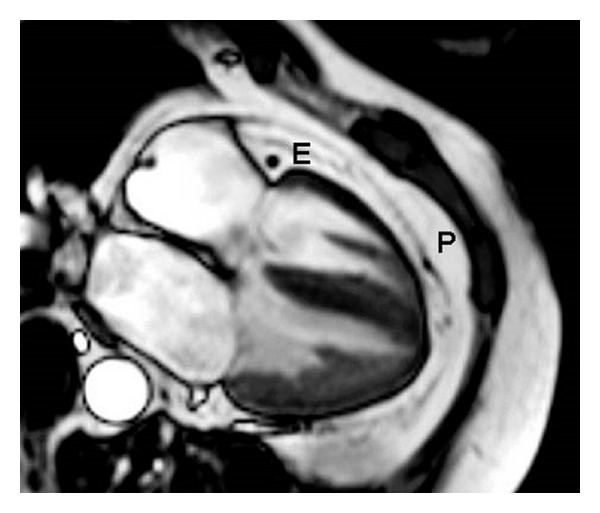
Four-chamber view of the heart. Four-chamber view of the heart where the signal from blood and muscle are suppressed. E: epicardial fat; P: pericardial fat.

Pericardial fat is the adipose tissue surrounding the heart. It consists of two layers: epicardial fat (visceral fat) originating from mesothelial cells and paracardial or mediastinal fat, originating from mesenchymal cells [83] (Figure 3).

Around 80% of the heart is surrounded by epicardial fat and epicardial fat makes out 20% of total heart weight. The latter varies more among men than in women; 15.2–25.2% and 19.5–21.7%, respectively. The largest part can be found around the right ventricle followed by the anterior wall. The amount of epicardial fat increases with age until age 20–40, thereafter there is no age dependence [83, 84].

Several functions have been proposed for epicardial fat tissue [84]. Scientific proof is, however, rather difficult to obtain since most animal species have very little epicardial fat [85]. In guinea pigs, rates of lipolysis and lipogenesis were 2 fold higher in epicardial fat than in other fat depots. This led to the assumption that epicardial fat might act as a buffer to protect the myocardium from highly toxic fatty acid levels and to provide fatty acids as a direct energy source in times of energy demand [85, 86]. Coronary arteries are embedded in epicardial fat so that another putative function might be to protect the coronary arteries from the tension and torsion induced by the arterial pulse wave and provide an environment in which the coronary arteries can easily expand. This fat compartment also acts as a metabolically active organ, secreting cytokines [87, 88].

Several cross-sectional studies have suggested a positive relation between an increased epicardial fat volume and coronary artery disease [89–92]. Furthermore, an increased epicardial fat volume has been associated with insulin resistance in nondiabetic obese patients [93] and with the presence of T2DM in a Han Chinese population [94, 95]. Therefore, it is interesting to study the effects of weight loss and diet on epicardial fat and insulin resistance.

### 7.1. Effect of Diet on Epicardial Fat

Two studies have examined the effect of diet-induced weight loss on epicardial fat. Kim et al. [96] studied 27 moderately obese NGT subjects, who lost 11% (9.5 kg) of weight during a 12-week weight loss intervention study. Epicardial fat thickness measured over the right ventricle wall by echocardiography decreased by 17% from baseline. Iacobellis et al. [97] studied 20 severely obese (BMI 45 ± 5 kg/m^2^) subjects (probably including patients with IGT or T2DM) who followed a 6-month low-calorie diet (900 kcal/day) and lost 20% (25 ± 10 kg) of bodyweight. Epicardial fat decreased by 32% from baseline. This was accompanied by an improvement in left ventricular mass and diastolic cardiac function. The change in diastolic function was also positively correlated with the change in epicardial fat thickness. Moreover, in 15 obese patients with T2DM, we found a significant decrease in pericardial fat after weight loss with a 16-week VLCD, measured with MRI (accepted for publication in Obesity) [98].

### 7.2. Effect of Exercise on Epicardial Fat

To date, only two studies examined the effect of (aerobic) exercise on epicardial fat [99, 100]. In one study, 24 obese NGT (BMI 30.7 ± 3.3 kg/m^2^) middle-aged Japanese men followed a supervised exercise program for 3 months. The exercise intensity was gradually increased in 4 weeks from 50–60 to 60–70% of the maximum heart rate 3 days/week 60 minutes, which was continued for the remainder of the study. Following the intervention the BMI decreased by 4.3 ± 3.0% (circa −1 kg/m^2^) and VO_2max⁡_ increased by 20%. Epicardial fat thickness measured by echocardiography over the free wall of the right ventricle decreased significantly. The change in visceral adipose tissue (−15%) was significantly correlated with the change in epicardial adipose tissue (−8.6%).

In the other study, 32 obese postmenopausal women were randomized to diet-only or diet combined with moderate or intensive exercise for 20 weeks. All three groups had a similar 15% reduction in bodyweight and a 17% reduction in pericardial fat. However, no differences were observed between the diet-only and diet with exercise group [100].

## 8. Myocardial Triglyceride Content

In addition to the epicardial/pericardial fat depositions, TG can also be stored within the cardiomyocytes. This is referred to as myocardial TG content or myocardial steatosis. Myocardial TG accumulation can be measured with great sensitivity by ^1^H-MRS [101]. Patients with IGT and T2DM have an increased myocardial TG content compared to obese and lean controls [102–104]. This accumulation of myocardial TG is a result of excessive fatty acid uptake relative to the oxidation. If fatty acids are converted to myocardial TG, several intermediates are released (e.g., ceramide) which in animal models caused cardiac dysfunction [105, 106]. In T2DM patients, myocardial steatosis was associated with impaired left ventricular diastolic function [104]. Indeed, an increased fatty acid uptake in the myocardium has been found in healthy obese and T2DM patients compared to healthy lean subjects [107, 108]. But in the one study that investigated the fate of the intracellular fatty acids in the resting state in insulin-naïve T2DM patients as compared to controls an increase, not a decrease in fatty acid oxidation was found. Fatty acid reesterification was negligible but lower in T2DM patients as compared to the controls [108]. Interestingly, neither plasma NEFA levels nor myocardial blood flow was increased both in subjects with obesity [107] and T2DM [108] suggesting another mechanism, for example, at the (cellular) level of the FAT/CD36, to account for the increased fatty acid uptake (Figure 2(c)).

The myocardial TG content is not static. Three days of severe caloric restriction (450 kcal/day to complete starvation) in healthy volunteers and patients with T2DM increases myocardial TG content, which is associated with a decrease in left ventricular diastolic function [109, 110].

### 8.1. Effect of Diet on Myocardial Triglyceride Content

Thirty-four obese (BMI 33.7 ± 0.7 kg/m^2^) healthy persons underwent a 6-week VLCD (550 kcal/day). Before and after the intervention intramyocardial TG were measured, a hyperinsulinaemic euglycaemic clamp was performed and either glucose uptake or fatty acid uptake was measured by positron emission tomography. The intervention led to a weight loss of 11.2 ± 0.6 kg and a nonsignificant decrease in myocardial TG content of 31%  (*n* = 8, *P* = 0.076). Myocardial fatty acid uptake decreased significantly. Myocardial mass and work decreased significantly by 7 and 26%, respectively [111].

Our group investigated the effect of a 16-week VLCD (450 kcal/day) on myocardial TG content in obese patients with T2DM [112]. We showed that a decrease in BMI (from 35.6 ± 1.2 to 27.5 ± 1.3 kg/m^2^) was associated with a significant decrease in myocardial TG content and an improvement in left ventricular diastolic function.

### 8.2. Effect of Exercise on Myocardial Triglyceride Content

In 14 NGT overweight-obese middle-aged men, a 12-week training program (3 times per week, combined aerobic and resistance training) reduced lipid accumulation at the cardiac septum (0.99 ± 0.15 to 0.54 ± 0.04%, *P* = 0.02) and improved left ventricular function slightly but significantly [113].

In obese patients with T2DM, however, a comparable 12-week intervention had no effect on cardiac lipid content measured in the septum, although both whole-body insulin sensitivity as well as left ventricular ejection fraction improved [114].

Together these data suggest that a decrease in cardiac lipid content is not a prerequisite for improved left ventricular function. Several factors might explain this. Firstly, a similar mechanism like with IMCLs in endurance-trained athletes (have increased IMCLs but an increased fatty acid oxidative capacity) might play a role. It would be, therefore, interesting to study endurance-trained athletes to see whether they have, like in skeletal muscle, higher levels of intramyocardial TG than healthy controls and whether this is associated with increased myocardial fatty acid oxidation and is related with cardiac function. On the other hand, improved insulin sensitivity with lower glucose and lipid levels might account for the improvement in cardiac function in the above 2 studies. However, plasma lipid levels did not change in the 2 studies.

## 9. Discussion

T2DM is a multifactorial disease in which genetic, environmental and lifestyle factors induce insulin resistance and impaired insulin secretion, ultimately leading to chronic hyperglycemia and its complications. Given the association between T2DM and obesity the recent focus of research has been the link between them. Vague already described a link between visceral adipose tissue, insulin resistance and T2DM in 1947 [115]. But it has not been until the start of the obesity and diabetic epidemic that further elaboration on his work started. This research revealed that adipose tissue is not merely a storage depot for TG but actively secretes a vast array of factors such as cytokines, metalloproteinases, and adipokines that can induce inflammation and insulin resistance [9].

In addition, with the advancement of radiological techniques it has become apparent that patients with insulin resistance and T2DM not only have a higher visceral to subcutaneous fat ratio as compared to healthy subjects but that TG are also stored in other organs called ectopic fat depositions, for example, in the liver, skeletal muscle, heart, and perhaps the pancreas [19, 116].

It has been proposed that genetic, environmental, and behavioral factors determine the response of adipose tissue to excess energy intake over energy expenditure. A physiologic response to this would be adipocyte hyperplasia, that is both an increase in adipocyte number with a small increase in adipocyte size. A pathologic response leads to adipocyte hypertrophy. Hypertrophic adipocytes become stressed because of hypoxia and nutritional overload of the endoplasmatic reticulum. Subsequently, stress and inflammatory pathways are activated. The adipocytes secrete cytokines and chemokines that interfere with the insulin signaling pathway. In addition, macrophages are attracted in the stromal vascular fraction of adipose tissue and sustain a chronic inflammatory response. Insulin-resistant hypertrophic adipocytes have increased lipolytic activity, together with the impaired ability of to take up FFAs, and consequently, a redirection of lipids towards nonadipose tissues (ectopic fat deposition) ensues [10].

If fatty acid beta-oxidation in the mitochondria cannot keep up with the increased supply of FFA to nonadipose tissues such as the liver, skeletal muscle, pancreas, and heart, accumulation of lipid intermediates like DAG and ceramides occurs. These lipid intermediates lead to activation of serine/threonine kinases that phosphorylate IRS molecules on serine residues. Serine phosphorylated IRS do not function properly, hence insulin signaling is impaired, and normal metabolic processes are disrupted.

Indeed, ectopic fat in the liver [67] and muscle [26] is positively correlated with insulin resistance and T2DM. Myocardial steatosis is associated with impaired diastolic function [104], and fat around the heart is associated with coronary artery disease [93] and related to whole-body insulin resistance [90]. The consequences of elevated fatty acids and/or lipid accumulation in the pancreas seem to be only in order when elevated glucose levels are already present [11].

The data in this paper show that substantial weight loss mobilizes ectopic fat stores in all organs and that this is associated with an improvement of the function of that organ. Thus, a reduction in hepatic TG was accompanied by a decline in fasting EGP [41, 73] and an improvement in the insulin suppressibility of EGP. A decrease in myocardial TG [112] and epicardial fat [97] were both associated with improved diastolic cardiac function. Finally, a decline in IMCLs leads to an improved insulin-stimulated glucose disposal [50].

It should be noted, however, that the amount of weight loss and/or the severity of caloric restriction are of influence on this positive effect and that there seems to be a tissue-specific reaction. For example, around eight kilograms weight loss following a 1200 kcal/day diet for 7 weeks led to a decrease in hepatic TG and improved insulin sensitivity of the liver but had no effect on insulin-stimulated glucose disposal or IMCLs in obese T2DM patients [41]. When obese women with a history of gestational diabetes were subdivided into groups with high and low liver fat, a similar weight loss led to greater loss of hepatic fat in the high liver fat group while both groups lost an equal amount of visceral and subcutaneous fat [72]. A VLCD for 8 weeks in moderately obese (BMI 33.6 ± 1.2 kg/m^2^) patients with T2DM < 4 years duration decreased body weight by 15.3 ± 1.2 kg. Like the study with 8 kg weight loss by hypocaloric diet, this decreased IHL content and improved hepatic insulin sensitivity but had no effect on insulin-stimulated glucose disposal. Apparently, either a longer duration of severe caloric restriction or a greater weight loss is necessary to improve insulin-stimulated glucose disposal. We showed that a prolonged VLCD in more severely obese insulin-dependent T2DM patients leading to around 22 kg of weight reduction improved insulin-stimulated glucose disposal and decreased IMCLs [50]. We recently corroborated these findings in a similar group of patients who underwent a 16-week VLCD (clamp data submitted for publication): ~27 kg weight loss improved both hepatic as well as peripheral insulin sensitivity. The largest reduction occurred in hepatic TG content (−85%), whereas IMCLs accumulation in the skeletal muscle decreased by 38%. The relative reduction in visceral fat was larger than the reduction in subcutaneous abdominal fat (−60% and −45% resp.) (accepted for publication in Obesity [98]).

The above studies suggest that hepatic TG content is the most easily mobilized, followed by visceral fat. The tissue-specific reaction to dietary interventions is also present when, vice versa, patients are subjected to high-fat feeding. Three days of a high-fat high-energy diet in health young males greatly increased hepatic TG stores but had no effect on myocardial TG [117].

 Few studies have investigated the effect of exercise per se, that is, exercise without weight loss and/or caloric restriction. The effect of exercise varies in the different organs with respect to TG accumulation. In muscle, exercise can even increase IMCLs [47, 55, 56]. However, when this is accompanied with increased fatty acid oxidation this is positive and in accordance with the athlete's paradox. The latter refers to the fact that endurance-trained athletes have increased IMCLs but are very insulin sensitive [26]. In these athletes the IMCLs are a substrate source during exercise, and the high turnover rate prevents accumulation of lipid intermediates that have a negative effect on insulin signaling and can form lipid peroxides. In the sedentary state, when metabolic flexibility is low, IMCLs accumulate with the afore-mentioned deleterious effect on cellular processes [27]. Exercise alone can either increase [47, 55, 56] or decrease IMCLs [54] but does improve insulin sensitivity (Table 1). Apart from a decrease in lipid intermediates with increased fatty acid oxidation, an increase in capillary density and activation of AMPK with subsequently enhanced GLUT4 translocation might also be involved in the observed improvement in peripheral insulin sensitivity. Exercise in combination with diet also depletes hepatic TG content and improves hepatic insulin sensitivity [78], whereas it is undecided yet whether exercise alone depletes hepatic TG content [80, 81]. The underlying mechanism for depletion of IHL is probably not direct but via a decrease in factors produced by adipose [9] and skeletal muscle tissue. Exercise only reduces epicardial fat, but cardiac function was not measured in that study [99]. Exercise improved left ventricular ejection fraction after a 12-week exercise intervention in obese NGT and obese T2DM subjects. However, myocardial TG content in the T2DM patients were not affected, suggesting that either a decrease in myocardial TG content is not relevant for the improvement in cardiac function, or that we might be looking to the same paradox found in skeletal muscle (athletes paradox [26]).

Further research should focus both on the origins and processes involved in adipose tissue dysfunction as well as on the consequences of ectopic fat on the cellular level. One of the key questions is whether the impaired fatty acid oxidation is the cause or consequence of impaired mitochondrial function. Recent evidence points to the latter [38]. That might have consequences for therapy. In addition, the role of the gut microbiota in inducing inflammation and insulin resistance should be unraveled [118, 119]. Nevertheless, given the fact that subjects adopting a Western diet rapidly become insulin resistant [120], and that this diet both changes gut microbiota, inflammation, and adiposity (vice versa weight loss and healthy diets change these parameters for the better) strongly supports that excess dietary fat induces an inflammatory response and impairs mitochondrial function if lipid oxidation cannot keep up with increased FFA delivery. Diet and exercise are powerful tools in improving both ectopic fat deposition and the function of the organ in which the ectopic fat is deposited. Diet and lifestyle intervention, therefore, deserve more attention, both as preventive measure for obesity and T2DM as well as for the treatment of insulin resistance and T2DM.

When prescribing diet and exercise, it appears that exercise only has little effect on body weight and depletion of intracellular lipid stores but does improve skeletal muscle insulin sensitivity (most likely via increased contraction mediated GLUT-4 translocation) and perhaps cardiac function. With respect to diets only, moderate caloric restriction (>1200 kcal/day) and moderate weight loss (3–15 kg) only improve hepatic insulin sensitivity. More severe energy restriction (VLCD < 800 kcal/day) for a longer period (>8 weeks) and/or greater weight loss (>20 kg) are necessary to improve insulin-stimulated peripheral glucose uptake.

## Figures and Tables

**Table 1:** Effect of diet and exercise on insulin sensitivity and intramyocellular lipid (IMCLs) content.

Ref.	Patients	*n*	BMI start	Age	Intervention	Duration	Body weight loss	Effect on skeletal muscle	Effect on IMCLs
			kg/m^2^	yrs			kg	insulin sensitivity	
[45]	Obese NGT	12	31 ± 2	Unknown	25% caloric restriction	6 months	−8 kg	Si: no change	No change
		12	33 ± 2		12,5% caloric restriction + 12,5% exercise		−8 kg	Si 37 ± 18%, *P* < 0.01	No change
		11	33 ± 2		15% weight loss hypocaloric diet 1200 kcal/day		−11 kg	Si 70 ± 34%, *P* < 0.04	No change
		11	31 ± 2		controls		0 kg	Si: no change	No change

[39]	Morbid obese NGT	9	48 ± 9	39 ± 12	Diet 1200 kcal/day	6 months	−14 ± 12 kg	M value: no change	No change
		8	51 ± 8	39 ± 12	Biliopancreatic diversion		−33 ± 10 kg	M value 23 ± 3 to 52 ± 11 *μ*mol/kg FFM/min, *P* < 0.05	1.6 ± 1.1 to 0.2 ± 0.4 AU, *P* < 0.05
	Controls	7	27 ± 1	35 ± 11	Controls		Unknown	M value: no change; baseline 53 ±13 *μ*mol/kg FFM/min	No change; baseline 0.1 ± 0.2

[46]	Obese NGT	20	34 ± 1	42 ± 2	−700 kcal compared to normal diet	15 weeks	−11 kg	OGTT: no change	No change
					followed bij energy restriction + exercise	21 ± 2 weeks	−5 kg	OGTT: no change	No change

[41]	Obese T2DM	8	30 ± 1	47 ± 3	1200 kcal/day 3% fat diet (untill normoglycemia)	3–12 weeks (mean 7)		GDR: no change	No change
					studies after weight stabilisation period	4 weeks	−8 ± 1 kg		

[42]	Obese NGT	13	33 ± 2	Unknown	−522 kcal compared to normal diet	3 months	−6 kg	M value: no change	No change

[43]	Overweight T2DM	7	27 ± 3	55 ± 5	−25–30 kcal/kg LBM	2 weeks	BMI −1.5 ± 0.0%	M value: no change	No change
		7	27 ± 3	46 ± 3	−25–30 kcal/kg LBM + advice to walk 2-3 td 5-6 days/week		BMI −2.3 ± 0.1%	M value 5.3 ± 0.3 to 8.2 ± 0.5 mg/kg/min; *P* < 0.001	3.8 ± 0.4 to 3.1 ± 0.4 IMCL/Cr, *P* < 0.03

[44]	Morbid obese NGT	7	44 ± 6	Unknown	Diet 1200 kcal/day	6 months	−5 ± 4 kg	M value: no change	No change

[48]	Obese T2DM	13	36 ± 1	50 ± 3	VLCD 600–800 kcal/day	8 weeks	−9 ± 1 kg	HOMA-IR −0.9 is −44 ± 7%, *P* < 0.001	No change

[49]	Obese NGT	5	36 ± 5	38 ± 12	VLCD 700 kcal/day	6 days	−2.3 kg	GDR: no change	−56%, *P* = 0.006 (^1^H-MRS)
	Obese T2DM	7	37 ± 7	43 ± 6	VLCD 700 kcal/day	6 days	−3.7 kg	GDR: no change	−40%, *P* = 0.04 (^1^H-MRS)

[50]	Obese T2DM	10	40 ± 2	55 ± 3	VLCD 500 kcal/day until 50% excess weight was lost	Mean 17 weeks	−22 kg	GDR 18.8 ± 2.0 to 39.1 ± 2.8 umol/kg LBM/min, *P* < 0.001	7 ± 14 to 4 ± 1 AU, *P* < 0.002

[51]	Obese NGT	7	33 ± 1	46 ± 2	25% caloric restriction; goal 7% weight loss	18.6 ± 0.7 weeks	−11 ± 2%, ca −8 kg	M value increased 29 ± 7% *P* < 0.05	Decreased, *P* < 0.05
		9	35 ± 1	42 ± 3	25% caloric restriction +3/5 days/wk exercise at 60–70% MHR	19.2 ± 0.4 weeks	−9 ± 1%, ca −9 kg	M value increased 38 ± 9% *P* < 0.05	No change

[52]	Obese T2DM	10	34 ± 1	44 ± 3	25% caloric restriction +3/5 days/wk exercise at 60–70% MHR	16–20 weeks	−7.1 ± 0.1% = ca 7 kg	GDR 4.1 ± 0.6 to 6.3 ± 0.9 mg/kg LBM/min, *P* < 0.05	48 ± 1 to 50 ± 1 HU, *P* < 0.01 (CT)

[53]	Obese NGT	21	33	40	−500–1000 kcal compared to normal diet with 4–6 x/wk exercise at 65–75% MHR	16 weeks	−10 kg	GDR 6.5 naar 9.7 mg/kg LBM/min *P* < 0.05	No change

[54]	Obese IGT	11	34 ± 1	67 ± 1	−600 kcal/day compared to normal diet and aerobic exercise 5 days/wk 60 min at 75% VO_2max⁡_	12 weeks	ca −8 kg	M value 2.9 ± 0.3 to 4.7 ± 0.6 mg/kg LBM/min, *P* < 0.01	3.9 ± 0.6 to 2.5 ± 0.3 LAI%, *P* < 0.05
		12	35 ± 2	66 ± 1	Aerobic exercise 5 days/wk 60 min at 75% VO_2max⁡_	12 weeks	ca −3 kg	M value 3.0 ± 0.4 to 4.2 ± 0.7 mg/kg LBM/min, *P* < 0.05	3.9 ± 0.6 to 3.0 ± 0.4 LAI%, *P* < 0.05

[55]	Obese NGT	25	30 ± 1	66 ± 1	4-5 days/wk supervised aerobic exercise at 75% MHR	16 weeks	−1.3 kg	M value: no change	21% increase, *P* < 0.01

[56]	Obese NGT	20	30 ± 1	59 ± 1	2x/week 30 min aerobic + 1x/week resistance exercise both at 55% VO_2max⁡_	12 weeks	no change	GDR: no change	No change
	Obese T2DM	18	30 ± 1	59 ± 1			no change	GDR 18.4 ± 1.4 to 21.0 ± 1.4 umol/kg/min, *P* < 0.05	No change

[47]	Obese IFG or IGT	8 8	31.2 ± 1.2 30.0 ± 1.0	66.9 ± 1.7 68.4 ± 1.5	Diet aimed at 10% weight loss and <30% fat Ex. 4-5 days/week 45 min/session moderate intense	16 weeks 16 weeks	−8.5 ± 1.5% −1.8 ± 0.9%	insulin-stimulated Rd + 20.6 ± 4.8%, from ~8 mg/kg FFM/min insulin-stimulated Rd +19.2 ± 12.9%, from ~8 mg/kg FFM/min	Decrease 16.0 ± 3.2% Increase 40.8 ± 18.2%

NGT: normal glucose tolerant; GDR: glucose disposal rate; HOMA-IR: homeostatic model assessment of insulin resistance; IGT: impaired glucose tolerant; LBM: lean body mass; T2DM: type 2 diabetes mellitus; AU: arbitrary units; LAI: mean percentage area of an individual skeletal muscle fibre that stains for lipids.

## Appendix

A. Literature Search Details
A.1. Pubmed (“Diabetes Mellitus, Type 2” [Majr] OR ((diabetic [ti] OR “diabetes mellitus” [ti]) AND “type 2” [ti]) OR “obesity” [Majr] OR “insulin resistance” [Majr] OR “obesity” [ti] OR “insulin resistance” [ti] OR obese [ti] OR “Non-Insulin-Dependent Diabetes Mellitus” [ti] OR “Type 2 Diabetes Mellitus” [ti] OR “Stable Diabetes Mellitus” [ti] OR “Maturity-Onset Diabetes Mellitus” [ti] OR MODY [ti] OR NIDDM [ti] OR “Adult-Onset Diabetes Mellitus” [ti]) AND “Weight Loss” [Mesh] OR “weight reduction” [tw] OR “weight loss” [tw] OR “Diet” [mesh] OR diet [tw] OR diets [tw] OR “Carbohydrate-Restricted” [tw] OR “Fat-Restricted” [tw] OR “Caloric Restriction” [tw] OR “Caloric Restrictions” [tw] OR “low carb” [tw] OR “low fat” [tw] OR “low caloric” [tw] OR VLCD [tw] OR “low energy” [tw] OR “low calorie” [tw] OR “Diet Therapy” [Mesh] OR “Behavior Therapy” [Mesh] OR “behavior therapy” [tw] OR “behaviour therapy” [tw] OR “conditioning therapy” [tw] OR “behavior modification” [tw] OR “behavior modifications” [tw] OR “cognitive therapy” [tw] OR “behaviour modification” [tw] OR “behaviour modifications” [tw] OR “Exercise” [mesh] OR exercise [tw] OR “Sports” [Mesh] OR sports [tw] OR sport [tw] AND (“ectopic fat” [tw] OR “ectopic lipid” [tw] OR “ectopic lipids” [tw] OR “hepatic steatosis” [tw] OR “fatty liver” [mesh] OR “fatty liver” [tw] OR NAFLD [tw] OR NASH [tiab] OR “nonalcoholic steatohepatitis” [tw] OR “nonalcoholic steatohepatitis” [tw] OR “liver steatosis” [tw] OR “hepatic triglyceride” [tw] OR “hepatic triglycerides” [tw] OR “liver triglyceride” [tw] OR “hepatic triglycerides” [tw] OR “Myocardial triglyceride” [tw] OR “Myocardial triglycerides” [tw] OR “Epicardial fat” [tw] OR “pericardial fat” [tw] OR “mediastinal fat” [tw] OR “intrathoracic fat” [tw] OR “intramyocellular triglyceride” [tw] OR “intramyocellular triglycerides” [tw] OR “intramyocellular lipid” [tw] OR “intramyocellular lipid” [tw] OR “intramyocellular fat” [tw] OR IMCLs [tw]).
A.2. EMBASE (OVID-Version) (*non insulin dependent diabetes mellitus/OR ((diabetic OR “diabetes mellitus”) AND “type 2”).ti OR exp *obesity/OR *insulin resistance/OR obesity.ti OR insulin resistance.ti OR obese.ti OR Non-Insulin-Dependent Diabetes Mellitus.ti OR “Type 2 Diabetes”.ti OR “Stable Diabetes Mellitus”.ti OR “Maturity-Onset Diabetes Mellitus”.ti OR MODY.ti OR NIDDM.ti OR “Adult-Onset Diabetes Mellitus”.ti) AND (weight reduction/OR weight reduction.mp OR weight loss.mp OR exp diet/OR diet.mp OR diets.mp OR exp diet therapy/OR exp dietary intake/OR Carbohydrate Restrict*.mp OR Fat Restrict*.mp OR Caloric Restrict*.mp OR low carb.mp OR low fat.mp OR low caloric.mp OR VLCD.mp OR low energy.mp OR low calorie.mp OR behavior therapy/OR behavior therapy.mp OR behaviour therapy.mp OR conditioning therapy.mp OR behavior modification/OR behavior modification*.mp OR cognitive therapy/OR psychotherapy/OR cognitive therapy.mp OR behaviour modification*.mp OR exp exercise/OR exercise.mp OR exp Sport/OR exp physical activity/OR sports.mp OR sport.mp AND (ectopic fat*.mp OR ectopic lipid*.mp OR exp fatty liver/OR hepatic steatosis.mp OR fatty liver.mp OR NAFLD.mp OR NASH.mp OR nonalcoholic steatohepatitis.mp OR nonalcoholic steatohepatitis.mp OR liver steatosis.mp OR hepatic triglycerid*.mp OR liver triglycerid* OR myocardial triglycerid*.mp OR epicardial fat.mp OR pericardial fat*.mp OR mediastinal fat*.mp OR intrathoracic fat*.mp OR intramyocellular triglycerid*.mp OR intramyocellular lipid*.mp OR intramyocellular fat*.mp OR IMCLs.mp).
A.3. Web of Science TI = (non insulin dependent diabetes mellitus OR ((diabetic OR “diabetes mellitus”) AND “type 2”) OR obesity OR insulin resistance OR insulin resistance OR obese OR Non-Insulin-Dependent Diabetes Mellitus OR “Type 2 Diabetes” OR “Stable Diabetes Mellitus” OR “Maturity-Onset Diabetes Mellitus” OR MODY OR NIDDM OR “Adult-Onset Diabetes Mellitus”) AND TS = (weight reduction OR weight loss OR diet OR diets OR dietary intake OR Carbohydrate Restrict* OR Fat Restrict* OR Caloric Restrict* OR low carb OR low fat OR low caloric OR VLCD OR low energy OR low calorie OR behavior therapy OR behaviour therapy OR conditioning therapy OR behavior modification* OR cognitive therapy OR psychotherapy OR behaviour modification* OR exercise OR physical activity OR sports OR sport) AND TS = (“ectopic fat*” OR “ectopic lipid*” OR “fatty liver” OR “hepatic steatosis” OR NAFLD OR NASH OR “nonalcoholic steatohepatitis” OR “nonalcoholic steatohepatitis” OR “liver steatosis” OR “hepatic triglycerid*” OR “liver triglycerid*” OR “myocardial triglycerid*” OR “epicardial fat” OR “pericardial fat*” OR “mediastinal fat*” OR “intrathoracic fat*” OR “intramyocellular triglycerid*” OR “intramyocellular lipid*” OR “intramyocellular fat*” OR IMCLs).
A.4. Cochrane Library

ID Search
#1 MeSH descriptor Diabetes Mellitus, Type 2 explode all trees
#2 MeSH descriptor Obesity explode all trees
#3 MeSH descriptor Insulin Resistance explode all trees
#4 (diabet* AND “type 2”) OR obesity OR insulin resistance OR obese OR “Non-Insulin-Dependent Diabetes Mellitus” OR “Type 2 Diabetes Mellitus” OR “Stable Diabetes Mellitus” OR “Maturity-Onset Diabetes Mellitus” OR MODY OR NIDDM OR “Adult-Onset Diabetes Mellitus”:ti
#5 (#1 OR #2 OR #3 OR #4)
#6 MeSH descriptor Weight Loss explode all trees
#7 MeSH descriptor Diet explode all trees
#8 MeSH descriptor Diet Therapy explode all trees
#9 MeSH descriptor Behavior Therapy explode all trees
#10 MeSH descriptor Exercise explode all trees
#11 MeSH descriptor Sports explode all trees
#12 ((“anti obesity” OR “anti-obesity” OR antiobesity OR “weight-loss” OR “weight loss”) AND “weight reduction” OR “weight loss” OR diet OR diets OR “Carbohydrate-Restricted” OR “Fat-Restricted” OR “Caloric Restriction” OR “Caloric Restrictions” OR “low carb” OR “low fat” OR “low caloric” OR VLCD OR “low energy” OR “low calorie” OR “behavior therapy” OR “behaviour therapy” OR “conditioning therapy” OR “behavior modification” OR “behavior modifications” OR “cognitive therapy” OR “behaviour modification” OR “behaviour modifications” OR exercise OR sports OR sport *:ti,ab,kw
#13 (#6 OR #7 OR #8 OR #9 OR #10 OR #11 OR #12)
#14 MeSH descriptor Fatty Liver explode all trees
#15 (“ectopic fat” OR “ectopic lipid” OR “ectopic lipids” OR “hepatic steatosis” OR “fatty liver” OR NAFLD OR NASH OR “nonalcoholic steatohepatitis” OR “nonalcoholic steatohepatitis” OR “liver steatosis” OR “hepatic triglyceride” OR “hepatic triglycerides” OR “liver triglyceride” OR “hepatic triglycerides” OR “Myocardial triglyceride” OR “Myocardial triglycerides” OR “Epicardial fat” OR “pericardial fat” OR “mediastinal fat” OR “intrathoracic fat” OR “intramyocellular triglyceride” OR “intramyocellular triglycerides” OR “intramyocellular lipid” OR “intramyocellular lipid” OR “intramyocellular fat” OR IMCLs): ti,ab,kw
#16 (#14 OR #15)
#17 (#5 AND #13 AND #16).

## Abbreviations

ALT: Alanine aminotransferase
AMPK: Activate adenosine monophosphate-activated protein kinase
AST: Aspartate aminotransferase
BMI: Body mass index
CPT1: Carnitine-palmitoyl transferase 1
CR: Calorie restriction
CT: Computertomography
CVD: Cardiovascular disease
DAG: Diacylglycerol
EGP: Endogenous glucose production
ER: Endoplasmatic reticulum
FAT/CD36: Fatty acid transporter CD36
FATP: Fatty acid transport protein
FABP: Fatty acid binding protein
FFA: Free fatty acid
FOXO: Forkhead box protein O
GLUT4: Glucose transporter 4
IGT: Impaired glucose tolerant
IL: Interleukin
IHL: Intrahepatic lipids
IMCLs: Intramyocellular lipid content
IRS1: Insulin receptor substrate 1
^1^H-MRS: Proton magnetic resonance spectroscopy
HOMA-IR: Homeostatic model assessment of insulin resistance
LC-CoA: Long-chain acyl-CoA
MRI: Magnetic resonance imaging
NAFLD: Nonalcoholic fatty liver disease
NASH: Nonalcoholic steatohepatitis
NGT: Normal glucose tolerant
PI3K: Phosphatidylinositol 3-kinase
PKB: Protein kinase B
PKC: Protein kinase C
T2DM: Type 2 diabetes mellitus
TNF*α*: Tumour necrosis factor alpha
TG: Triglyceride
UPR: Unfolded protein response
VLCD: Very low-calorie diet
VO_2max⁡_: Maximum aerobic capacity.
